# Redetermination of 1,3-diammonio-1,2,3-tride­oxy-*cis*-inositol dichloride

**DOI:** 10.1107/S1600536812012366

**Published:** 2012-04-18

**Authors:** Christian Neis, Günter J. Merten, Philipp Altenhofer, Kaspar Hegetschweiler

**Affiliations:** a Fachrichtung Chemie, Universität des Saarlandes, Postfach 151150, D-66041 Saarbrücken, Germany

## Abstract

The crystal structure of the title compound, C_6_H_16_N_2_O_3_^2+^·2Cl^−^, has been reported previously by Palm [*Acta Cryst.* (1967 ▶), **22**, 209–216] from Weisenberg camera data, with *R*1 = 10.5%, isotropic refinement of non-H atoms and H atoms not located. We remeasured a data set of the title compound and present a more precise structure determination. The asymmetric unit contains two unique 1,3-diammonio-1,2,3-tride­oxy-*cis*-inositol cations and four Cl^−^ counter-ions. The cyclo­hexane rings of both inositol cations adopt chair conformations with two axial hy­droxy groups. An extended network of hydrogen bonds is formed. The four chloride counter ions are hydrogen bonded to the hydroxy and ammonium groups of the cations by N—H⋯Cl and O—H⋯Cl interactions. The cations are aligned into wavy layers by cation⋯cation interactions of the form N—H⋯O(ax), N—H⋯O(eq) and O(ax)—H⋯O(eq). Intramolecular hydrogen bonding between the axial hydroxy groups is, however, not observed.

## Related literature

An earlier, less accurate structure determination of the title compound was performed by Palm (1967 ▶). The crystal structure of 1,3-diammonio-1,2,3-tride­oxy-*cis*-inositol sulfate has been reported by Neis *et al.* (2012 ▶). The importance of intra­molecular hydrogen bonding in 1,3,5-tris­ubstituted cyclo­hexane derivatives has been described by Gencheva *et al.* (2000 ▶), Saaidi *et al.* (2008 ▶) and Neis *et al.* (2010 ▶), and the implication of increased 1,3-diaxial repulsion on the conformation of a cyclo­hexane ring has been discussed by Fritsche-Lang *et al.* (1985 ▶), Kramer *et al.* (1998 ▶) and Kuppert *et al.* (2006 ▶). For the synthesis, see: Merten *et al.* (2012 ▶). For the treatment of hydrogen atoms in *SHELXL*, see: Müller *et al.* (2006 ▶).
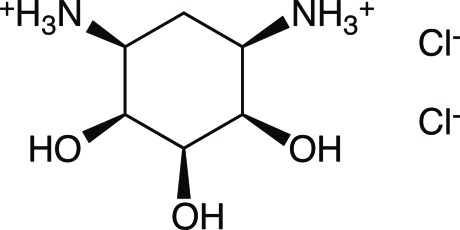


## Experimental

### 

#### Crystal data

C_6_H_16_N_2_O_3_^2+^·2Cl^−^
*M* *_r_* = 235.11
Monoclinic, 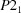

*a* = 7.7899 (4) Å
*b* = 10.1254 (5) Å
*c* = 13.0136 (7) Å
β = 91.156 (2)°
*V* = 1026.25 (9) Å^3^
*Z* = 4
Mo *K*α radiation
μ = 0.61 mm^−1^
*T* = 130 K
0.30 × 0.22 × 0.15 mm

#### Data collection

Bruker–Nonius X8 APEX KappaCCD diffractometer
Absorption correction: multi-scan (*SADABS*; Bruker, 2010 ▶) *T*_min_ = 0.838, *T*_max_ = 0.914
16242 measured reflections
4459 independent reflections
4429 reflections with *I* > 2σ(*I*)
*R*_int_ = 0.017

#### Refinement

*R*[*F* ^2^ > 2σ(*F* ^2^)] = 0.017
*wR*(*F* ^2^) = 0.047
*S* = 1.06
4459 reflections
289 parameters
19 restraints
H atoms treated by a mixture of independent and constrained refinement
Δρ_max_ = 0.27 e Å^−3^
Δρ_min_ = −0.15 e Å^−3^
Absolute structure: Flack (1983 ▶), 2093 Friedel pairs
Flack parameter: 0.01 (3)

### 

Data collection: *APEX2* (Bruker, 2010 ▶); cell refinement: *SAINT* (Bruker, 2010 ▶); data reduction: *SAINT*; program(s) used to solve structure: *SHELXS97* (Sheldrick, 2008 ▶); program(s) used to refine structure: *SHELXL97* (Sheldrick, 2008 ▶); molecular graphics: *DIAMOND* (Brandenburg, 2011 ▶); software used to prepare material for publication: *SHELXL97* and *PLATON* (Spek, 2009 ▶).

## Supplementary Material

Crystal structure: contains datablock(s) global, I. DOI: 10.1107/S1600536812012366/sj5213sup1.cif

Structure factors: contains datablock(s) I. DOI: 10.1107/S1600536812012366/sj5213Isup2.hkl

Additional supplementary materials:  crystallographic information; 3D view; checkCIF report

## Figures and Tables

**Table 1:** Hydrogen-bond geometry (Å, °)

*D*—H⋯*A*	*D*—H	H⋯*A*	*D*⋯*A*	*D*—H⋯*A*
N18—H18*A*⋯Cl4	0.89 (1)	2.28 (1)	3.1411 (11)	163 (2)
N18—H18*B*⋯Cl1^i^	0.90 (1)	2.37 (2)	3.2646 (12)	177 (2)
N18—H18*C*⋯Cl3^ii^	0.91 (1)	2.26 (1)	3.1634 (11)	175 (2)
N22—H22*C*⋯Cl4^iii^	0.87 (1)	2.21 (1)	3.0761 (11)	172 (2)
N22—H22*A*⋯Cl1	0.88 (2)	2.28 (2)	3.1466 (12)	168 (2)
N22—H22*B*⋯Cl3	0.87 (1)	2.32 (2)	3.1518 (12)	162 (2)
O19—H19⋯O9^iv^	0.81 (2)	1.91 (2)	2.7168 (13)	172 (2)
O20—H20⋯Cl2^v^	0.88 (2)	2.48 (2)	3.2220 (10)	143 (2)
O21—H21⋯Cl2	0.83 (1)	2.39 (2)	3.2096 (10)	174 (2)
N7—H7*C*⋯O20^vi^	0.89 (1)	2.05 (2)	2.8560 (15)	151 (2)
N7—H7*B*⋯O19	0.85 (1)	2.11 (2)	2.9404 (14)	164 (2)
N7—H7*A*⋯Cl3	0.89 (1)	2.70 (2)	3.4038 (11)	138 (1)
O8—H8⋯Cl2^vi^	0.84 (1)	2.29 (2)	3.1256 (10)	175 (2)
O9—H9⋯Cl1^iv^	0.80 (2)	2.27 (2)	3.0144 (10)	156 (2)
O10—H10⋯Cl4^vii^	0.85 (2)	2.14 (2)	2.9889 (10)	177 (2)
N11—H11*C*⋯Cl2^v^	0.89 (2)	2.37 (2)	3.2132 (12)	159 (2)
N11—H11*B*⋯Cl1^v^	0.86 (1)	2.51 (2)	3.3007 (11)	154 (2)
N11—H11*A*⋯Cl2	0.90 (2)	2.61 (2)	3.4618 (13)	158 (2)

Symmetry codes: (i) 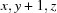; (ii) 
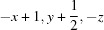
; (iii) 
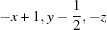
; (iv) 
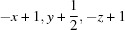
; (v) 
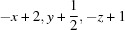
; (vi) 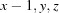; (vii) 
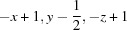
.

**Fig. 1.**
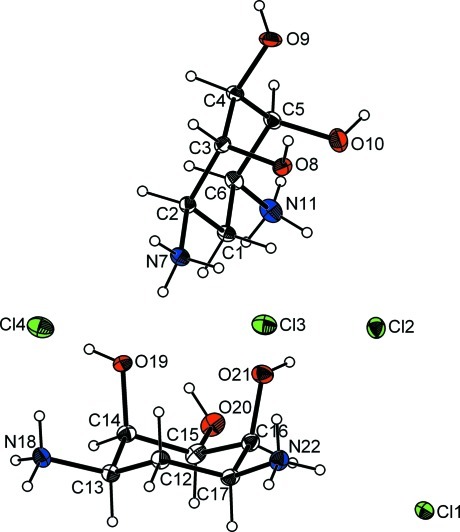
Molecular structure of the title compound. The asymmetric unit comprising two crystallographically independent cations and four crystallographically independent chloride counter ions is shown. Displacement ellipsoids are drawn at the 50% probability level.

**Fig. 2.**
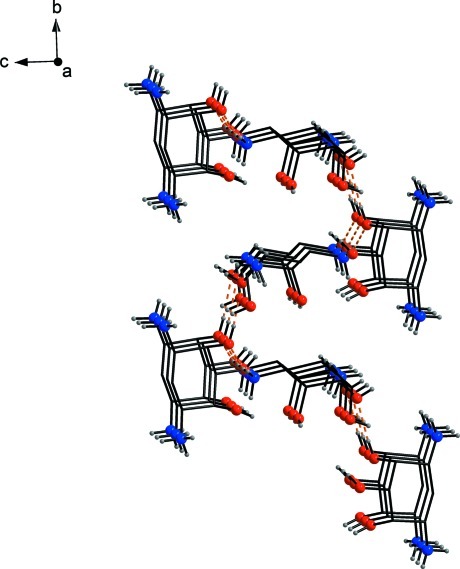
Section of the puckered layer, which is formed by hydrogen bonding between cation 1 and cation 2 entities (ball and stick model).

## supplementary crystallographic information

### Comment

The title compound contains two independent 1,3-diammonio-1,2,3-trideoxy-*cis*-inositol dications (denoted as cation 1 and cation 2). As already noted by Palm (1967), the cyclohexane rings of both cations adopt a chair conformation, having one hydroxy and two ammonium groups in equatorial and two hydroxy groups in axial position. The puckering parameters of the two cyclohexane rings are: Q = 0.59 Å, θ = 4.3 °, φ = 337.8 ° (cation 1); Q = 0.58 Å, θ = 5.0 °, φ = 87.0 ° (cation 2). The cations are aligned to wavy layers, oriented parallel to the *ab* plane, by direct cation 1···cation 2 interactions of the form N—H···O(ax), N—H···O(eq) and O(ax)—H···O(eq). In these layers, each cation of one type is interlinked with three cations of the other. In addition, cation 1···cation 1 interactions are formed by weak C—H···O hydrogen bonds between the axial oxygen atoms and two axial C—H hydrogen atoms of a neighbour (O···C distances: 3.277 and 3.215 Å, O···H distances: 2.470 and 2.485 Å, O···H—C angles 137 and 129 °). Cation 2···cation 2 hydrogen bonding is not observed. The four chloride counter ions are also involved in the extended hydrogen bonding network. Considering Cl···H distances up to 2.7 Å, Cl1 has a coordination number of four (irregular geometry). The coordination number of Cl2 is 5 and its geometry lies closer to a tetragonal pyramid than to a trigonal bipyramid (τ = 1/4). Cl3 and Cl4 have coordination numbers of three. The geometry is again irregular. All O—H and N—H groups in the two cations act as hydrogen donors, however, not all of the axial hydroxy groups act as hydrogen acceptors (if the abovementioned, weak C—H···O contacts are disregarded). This observation is in agreement with the well established concept that axial substituents are sterically more encumbered. In particular, it is of interest that no intramolecular O—H···O hydrogen bonding has been found for the axial hydroxy groups, although the corresponding O···O separations fall in the almost ideal range of 2.893 - 2.906 Å. For corresponding structures with three axial hydroxy or amino groups in a *syn*-1,3,5-triaxial arrangement, a different behaviour has frequently been noted (Gencheva *et al.*, 2000; Saaidi *et al.*, 2008; Neis *et al.*, 2010). If a third *syn*-axial substituent is present, it appears that formation of such intramolecular hydrogen bonds is sometimes even a prerequisite for the adoption of a stable cyclohexane chair. Disabling of hydrogen bonding (for instance by protonation of an amino group or by converting it into an amide) enforces the structure to escape the increasing repulsion by switching to a twisted boat conformation (Fritsche-Lang *et al.*, 1985; Kramer *et al.*, 1998; Kuppert *et al.*, 2006). It is thus noteworthy that in both cations of the title compound, such intramolecular O—H···O stabilisation is not required. Also in a corresponding sulphate salt (Neis *et al.*, 2012) no intramolecular hydrogen bonding between the two axial hydroxy groups has been found.

### Experimental

The title compound was prepared following the protocol given by Merten *et al.* (2012). ^1^H-NMR (D_2_O, pH 1.0): δ (p.p.m.) = 4.17 (2*H*), 3.74 (1*H*), 3.56 (2*H*), 2.12 (2*H*). ^13^C-NMR (D_2_O, pH 1.0): δ (p.p.m.) = 25.2, 43.0, 63.7, 68.2. Single crystals were grown from an aqueous solution by slow evaporation at 298 K.

### Refinement

All non-hydrogen atoms were refined using anisotropic displacement parameters. Hydrogen atoms were treated as recommended by Müller *et al.* (2006): A riding model was used for C-bonded hydrogen atoms. The positional parameters of the O- and N-bonded hydrogen atoms were refined using isotropic displacement parameters which were set to 1.5×*U*_eq_ of the pivot atom. In addition, restraints of 0.84 and 0.88 Å were used for the O—H and N—H distances, respectively.

### Crystal data

**Table:**

C_6_H_16_N_2_O_3_^2+^·2Cl^−^	*F*(000) = 496
*M**_r_* = 235.11	*D*_x_ = 1.522 Mg m^−^^3^
Monoclinic, *P*2_1_	Mo *K*α radiation, λ = 0.71073 Å
Hall symbol: P 2yb	Cell parameters from 9336 reflections
*a* = 7.7899 (4) Å	θ = 2.6–40.5°
*b* = 10.1254 (5) Å	µ = 0.61 mm^−^^1^
*c* = 13.0136 (7) Å	*T* = 130 K
β = 91.156 (2)°	Prism, colorless
*V* = 1026.25 (9) Å^3^	0.30 × 0.22 × 0.15 mm
*Z* = 4	

### Data collection

**Table:**

Bruker–Nonius X8 APEX KappaCCD diffractometer	4459 independent reflections
Radiation source: fine-focus sealed tube	4429 reflections with *I* > 2σ(*I*)
Graphite monochromator	*R*_int_ = 0.017
φ and ω scans	θ_max_ = 27.0°, θ_min_ = 1.6°
Absorption correction: multi-scan (*SADABS*; Bruker, 2010)	*h* = −9→9
*T*_min_ = 0.838, *T*_max_ = 0.914	*k* = −12→12
16242 measured reflections	*l* = −16→16

### Refinement

**Table:**

Refinement on *F*^2^	Secondary atom site location: difference Fourier map
Least-squares matrix: full	Hydrogen site location: inferred from neighbouring sites
*R*[*F*^2^ > 2σ(*F*^2^)] = 0.017	H atoms treated by a mixture of independent and constrained refinement
*wR*(*F*^2^) = 0.047	*w* = 1/[σ^2^(*F*_o_^2^) + (0.0292*P*)^2^ + 0.1788*P*] where *P* = (*F*_o_^2^ + 2*F*_c_^2^)/3
*S* = 1.06	(Δ/σ)_max_ = 0.001
4459 reflections	Δρ_max_ = 0.27 e Å^−^^3^
289 parameters	Δρ_min_ = −0.15 e Å^−^^3^
19 restraints	Absolute structure: Flack (1983), 2093 Friedel pairs
Primary atom site location: structure-invariant direct methods	Flack parameter: 0.01 (3)

### Special details

**Table:**

Geometry. All e.s.d.'s (except the e.s.d. in the dihedral angle between two l.s. planes) are estimated using the full covariance matrix. The cell e.s.d.'s are taken into account individually in the estimation of e.s.d.'s in distances, angles and torsion angles; correlations between e.s.d.'s in cell parameters are only used when they are defined by crystal symmetry. An approximate (isotropic) treatment of cell e.s.d.'s is used for estimating e.s.d.'s involving l.s. planes.
Refinement. Refinement of *F*^2^ against ALL reflections. The weighted *R*-factor *wR* and goodness of fit *S* are based on *F*^2^, conventional *R*-factors *R* are based on *F*, with *F* set to zero for negative *F*^2^. The threshold expression of *F*^2^ > σ(*F*^2^) is used only for calculating *R*-factors(gt) *etc*. and is not relevant to the choice of reflections for refinement. *R*-factors based on *F*^2^ are statistically about twice as large as those based on *F*, and *R*- factors based on ALL data will be even larger.

### Fractional atomic coordinates and isotropic or equivalent isotropic displacement parameters (Å^2^)

**Table:**

	*x*	*y*	*z*	*U*_iso_*/*U*_eq_	
C1	0.65695 (15)	0.78053 (13)	0.49259 (9)	0.0116 (2)	
H1A	0.7440	0.8297	0.4535	0.014*	
H1B	0.6742	0.6850	0.4805	0.014*	
C2	0.47789 (15)	0.82069 (11)	0.45618 (9)	0.0109 (2)	
H2	0.4629	0.9177	0.4672	0.013*	
C3	0.33980 (16)	0.74645 (11)	0.51435 (9)	0.0117 (2)	
H3	0.2245	0.7822	0.4936	0.014*	
C4	0.36884 (16)	0.76840 (13)	0.63009 (9)	0.0135 (2)	
H4	0.3478	0.8638	0.6453	0.016*	
C5	0.54882 (17)	0.73308 (12)	0.66995 (9)	0.0141 (2)	
H5	0.5605	0.7597	0.7438	0.017*	
C6	0.67808 (15)	0.81082 (12)	0.60699 (9)	0.0129 (2)	
H6	0.6605	0.9075	0.6185	0.015*	
N18	0.72743 (14)	1.01783 (11)	0.05624 (8)	0.0149 (2)	
H18A	0.6277 (19)	1.0191 (18)	0.0889 (12)	0.022*	
H18B	0.789 (2)	1.0895 (16)	0.0746 (14)	0.022*	
H18C	0.706 (2)	1.0188 (19)	−0.0129 (10)	0.022*	
N22	0.73895 (14)	0.53468 (11)	0.10007 (8)	0.0154 (2)	
H22C	0.706 (2)	0.5268 (19)	0.0357 (11)	0.023*	
H22A	0.801 (2)	0.4651 (17)	0.1183 (13)	0.023*	
H22B	0.646 (2)	0.5385 (19)	0.1356 (13)	0.023*	
O19	0.75839 (12)	0.93190 (9)	0.26803 (7)	0.01429 (18)	
H19	0.751 (2)	1.0080 (15)	0.2863 (13)	0.021*	
O20	1.09187 (12)	0.81557 (10)	0.32436 (7)	0.0206 (2)	
H20	1.028 (2)	0.8689 (19)	0.3606 (14)	0.031*	
O21	0.78841 (12)	0.64823 (10)	0.28780 (7)	0.01674 (19)	
H21	0.837 (2)	0.6185 (18)	0.3397 (12)	0.025*	
N7	0.45629 (14)	0.79085 (11)	0.34387 (8)	0.0132 (2)	
H7C	0.3542 (19)	0.8197 (17)	0.3215 (13)	0.020*	
H7B	0.537 (2)	0.8271 (17)	0.3103 (13)	0.020*	
H7A	0.462 (2)	0.7050 (14)	0.3315 (13)	0.020*	
C13	0.83453 (16)	0.90192 (12)	0.08871 (9)	0.0140 (2)	
H13	0.9361	0.8970	0.0431	0.017*	
O8	0.34834 (12)	0.61091 (9)	0.48626 (7)	0.01400 (17)	
H8	0.2471 (19)	0.5838 (18)	0.4902 (14)	0.021*	
C14	0.89926 (16)	0.92022 (12)	0.19938 (9)	0.0131 (2)	
H14	0.9704	1.0023	0.2037	0.016*	
O9	0.24058 (12)	0.69200 (10)	0.68068 (7)	0.01881 (19)	
H9	0.213 (2)	0.7301 (18)	0.7312 (13)	0.028*	
C15	1.01194 (16)	0.80171 (13)	0.22624 (9)	0.0152 (2)	
H15	1.1064	0.8011	0.1754	0.018*	
O10	0.58596 (14)	0.59647 (10)	0.66089 (7)	0.0204 (2)	
H10	0.601 (2)	0.5671 (19)	0.7219 (12)	0.031*	
C16	0.92115 (16)	0.66793 (12)	0.21605 (9)	0.0134 (2)	
H16	1.0084	0.5963	0.2255	0.016*	
N11	0.85723 (14)	0.77409 (13)	0.63986 (8)	0.0182 (2)	
H11C	0.931 (2)	0.8269 (18)	0.6089 (14)	0.027*	
H11B	0.872 (2)	0.780 (2)	0.7055 (11)	0.027*	
H11A	0.883 (2)	0.6931 (16)	0.6164 (14)	0.027*	
C17	0.84314 (16)	0.65722 (12)	0.10753 (9)	0.0132 (2)	
H17	0.9392	0.6500	0.0580	0.016*	
C12	0.73148 (16)	0.77507 (13)	0.07590 (9)	0.0137 (2)	
H12A	0.6929	0.7652	0.0033	0.016*	
H12B	0.6285	0.7786	0.1192	0.016*	
Cl1	0.95255 (4)	0.27346 (3)	0.13119 (2)	0.01574 (7)	
Cl2	0.96850 (3)	0.51134 (3)	0.48456 (2)	0.01623 (6)	
Cl3	0.36075 (4)	0.53635 (3)	0.18167 (2)	0.02006 (7)	
Cl4	0.34728 (4)	0.98731 (3)	0.12868 (2)	0.02147 (7)	

### Atomic displacement parameters (Å^2^)

**Table:**

	*U*^11^	*U*^22^	*U*^33^	*U*^12^	*U*^13^	*U*^23^
C1	0.0106 (5)	0.0148 (6)	0.0092 (5)	−0.0009 (4)	−0.0005 (4)	0.0008 (4)
C2	0.0121 (5)	0.0109 (5)	0.0096 (5)	−0.0002 (4)	−0.0004 (4)	−0.0002 (4)
C3	0.0111 (5)	0.0103 (5)	0.0138 (5)	0.0004 (4)	0.0021 (4)	−0.0004 (4)
C4	0.0166 (6)	0.0108 (5)	0.0134 (5)	−0.0016 (4)	0.0059 (4)	−0.0001 (4)
C5	0.0203 (6)	0.0122 (5)	0.0097 (5)	−0.0014 (5)	0.0021 (4)	0.0003 (4)
C6	0.0138 (6)	0.0140 (6)	0.0107 (5)	−0.0020 (4)	−0.0015 (4)	0.0004 (4)
N18	0.0175 (5)	0.0141 (5)	0.0131 (4)	−0.0007 (4)	−0.0003 (4)	0.0020 (4)
N22	0.0177 (5)	0.0142 (5)	0.0140 (5)	0.0010 (4)	−0.0013 (4)	−0.0019 (4)
O19	0.0168 (4)	0.0137 (4)	0.0125 (4)	0.0013 (3)	0.0033 (3)	−0.0020 (3)
O20	0.0189 (5)	0.0250 (5)	0.0176 (4)	0.0031 (4)	−0.0048 (4)	−0.0033 (4)
O21	0.0170 (5)	0.0219 (5)	0.0114 (4)	0.0020 (4)	0.0003 (3)	0.0017 (3)
N7	0.0133 (5)	0.0159 (5)	0.0104 (5)	−0.0016 (4)	−0.0012 (4)	0.0010 (4)
C13	0.0158 (6)	0.0136 (6)	0.0125 (5)	0.0003 (4)	0.0017 (4)	0.0005 (4)
O8	0.0129 (4)	0.0115 (4)	0.0177 (4)	−0.0025 (3)	0.0029 (3)	−0.0030 (3)
C14	0.0113 (5)	0.0139 (6)	0.0140 (6)	−0.0012 (4)	0.0011 (4)	−0.0014 (4)
O9	0.0214 (5)	0.0175 (5)	0.0180 (4)	−0.0036 (4)	0.0115 (4)	−0.0020 (4)
C15	0.0115 (5)	0.0173 (6)	0.0167 (6)	0.0015 (4)	−0.0028 (4)	−0.0033 (5)
O10	0.0302 (5)	0.0148 (4)	0.0162 (4)	0.0005 (4)	−0.0002 (4)	0.0018 (4)
C16	0.0128 (6)	0.0142 (6)	0.0133 (5)	0.0022 (4)	−0.0005 (4)	−0.0003 (4)
N11	0.0171 (5)	0.0222 (6)	0.0151 (5)	−0.0032 (5)	−0.0055 (4)	0.0027 (5)
C17	0.0135 (6)	0.0133 (6)	0.0127 (5)	0.0000 (4)	0.0021 (4)	−0.0020 (4)
C12	0.0166 (5)	0.0144 (6)	0.0101 (5)	0.0001 (5)	−0.0015 (4)	−0.0010 (4)
Cl1	0.01912 (14)	0.01465 (13)	0.01360 (12)	−0.00009 (11)	0.00369 (10)	0.00124 (10)
Cl2	0.01362 (13)	0.01454 (13)	0.02052 (14)	−0.00019 (10)	0.00009 (10)	0.00263 (11)
Cl3	0.01973 (14)	0.02535 (17)	0.01513 (13)	−0.00115 (12)	0.00145 (10)	−0.00093 (12)
Cl4	0.01848 (14)	0.03068 (18)	0.01520 (13)	−0.00287 (12)	−0.00074 (10)	0.00097 (12)

### Geometric parameters (Å, º)

**Table:**

C1—C2	1.5195 (16)	O19—H19	0.809 (15)
C1—C6	1.5257 (15)	O20—C15	1.4163 (15)
C1—H1A	0.9900	O20—H20	0.878 (15)
C1—H1B	0.9900	O21—C16	1.4212 (15)
C2—N7	1.4987 (14)	O21—H21	0.825 (14)
C2—C3	1.5258 (16)	N7—H7C	0.890 (14)
C2—H2	1.0000	N7—H7B	0.852 (14)
C3—O8	1.4222 (14)	N7—H7A	0.885 (14)
C3—C4	1.5347 (16)	C13—C12	1.5221 (18)
C3—H3	1.0000	C13—C14	1.5275 (16)
C4—O9	1.4341 (14)	C13—H13	1.0000
C4—C5	1.5275 (18)	O8—H8	0.838 (14)
C4—H4	1.0000	C14—C15	1.5232 (17)
C5—O10	1.4186 (15)	C14—H14	1.0000
C5—C6	1.5291 (16)	O9—H9	0.796 (15)
C5—H5	1.0000	C15—C16	1.5326 (17)
C6—N11	1.4983 (16)	C15—H15	1.0000
C6—H6	1.0000	O10—H10	0.854 (15)
N18—C13	1.4959 (16)	C16—C17	1.5299 (16)
N18—H18A	0.893 (14)	C16—H16	1.0000
N18—H18B	0.900 (14)	N11—H11C	0.885 (15)
N18—H18C	0.911 (13)	N11—H11B	0.862 (14)
N22—C17	1.4848 (16)	N11—H11A	0.898 (15)
N22—H22C	0.874 (14)	C17—C12	1.5283 (17)
N22—H22A	0.883 (15)	C17—H17	1.0000
N22—H22B	0.865 (14)	C12—H12A	0.9900
O19—C14	1.4339 (15)	C12—H12B	0.9900
			
C2—C1—C6	109.31 (10)	C2—N7—H7C	109.6 (11)
C2—C1—H1A	109.8	C2—N7—H7B	110.1 (12)
C6—C1—H1A	109.8	H7C—N7—H7B	110.5 (16)
C2—C1—H1B	109.8	C2—N7—H7A	111.7 (12)
C6—C1—H1B	109.8	H7C—N7—H7A	107.9 (17)
H1A—C1—H1B	108.3	H7B—N7—H7A	106.9 (17)
N7—C2—C1	109.54 (9)	N18—C13—C12	109.95 (10)
N7—C2—C3	108.55 (9)	N18—C13—C14	110.05 (10)
C1—C2—C3	111.43 (9)	C12—C13—C14	111.65 (10)
N7—C2—H2	109.1	N18—C13—H13	108.4
C1—C2—H2	109.1	C12—C13—H13	108.4
C3—C2—H2	109.1	C14—C13—H13	108.4
O8—C3—C2	108.11 (9)	C3—O8—H8	104.6 (12)
O8—C3—C4	112.66 (10)	O19—C14—C15	111.51 (10)
C2—C3—C4	108.91 (10)	O19—C14—C13	110.81 (10)
O8—C3—H3	109.0	C15—C14—C13	107.37 (10)
C2—C3—H3	109.0	O19—C14—H14	109.0
C4—C3—H3	109.0	C15—C14—H14	109.0
O9—C4—C5	111.16 (10)	C13—C14—H14	109.0
O9—C4—C3	106.39 (10)	C4—O9—H9	108.6 (14)
C5—C4—C3	114.55 (10)	O20—C15—C14	111.69 (10)
O9—C4—H4	108.2	O20—C15—C16	111.06 (10)
C5—C4—H4	108.2	C14—C15—C16	114.43 (10)
C3—C4—H4	108.2	O20—C15—H15	106.4
O10—C5—C4	112.79 (10)	C14—C15—H15	106.4
O10—C5—C6	108.65 (10)	C16—C15—H15	106.4
C4—C5—C6	107.90 (10)	C5—O10—H10	106.7 (13)
O10—C5—H5	109.1	O21—C16—C17	108.41 (10)
C4—C5—H5	109.1	O21—C16—C15	114.07 (10)
C6—C5—H5	109.1	C17—C16—C15	108.45 (10)
N11—C6—C1	108.07 (10)	O21—C16—H16	108.6
N11—C6—C5	109.81 (10)	C17—C16—H16	108.6
C1—C6—C5	111.11 (10)	C15—C16—H16	108.6
N11—C6—H6	109.3	C6—N11—H11C	109.0 (12)
C1—C6—H6	109.3	C6—N11—H11B	111.6 (13)
C5—C6—H6	109.3	H11C—N11—H11B	109.4 (18)
C13—N18—H18A	111.2 (12)	C6—N11—H11A	109.9 (12)
C13—N18—H18B	105.4 (12)	H11C—N11—H11A	104.3 (17)
H18A—N18—H18B	109.1 (17)	H11B—N11—H11A	112.3 (18)
C13—N18—H18C	112.1 (11)	N22—C17—C12	109.12 (10)
H18A—N18—H18C	109.2 (15)	N22—C17—C16	109.03 (10)
H18B—N18—H18C	109.8 (17)	C12—C17—C16	113.99 (10)
C17—N22—H22C	106.7 (12)	N22—C17—H17	108.2
C17—N22—H22A	110.8 (12)	C12—C17—H17	108.2
H22C—N22—H22A	109.4 (17)	C16—C17—H17	108.2
C17—N22—H22B	112.8 (13)	C13—C12—C17	109.47 (10)
H22C—N22—H22B	106.6 (16)	C13—C12—H12A	109.8
H22A—N22—H22B	110.3 (17)	C17—C12—H12A	109.8
C14—O19—H19	108.9 (13)	C13—C12—H12B	109.8
C15—O20—H20	107.5 (13)	C17—C12—H12B	109.8
C16—O21—H21	105.0 (13)	H12A—C12—H12B	108.2
			
C6—C1—C2—N7	−179.87 (10)	N18—C13—C14—O19	59.93 (13)
C6—C1—C2—C3	−59.74 (13)	C12—C13—C14—O19	−62.45 (13)
N7—C2—C3—O8	53.24 (12)	N18—C13—C14—C15	−178.07 (10)
C1—C2—C3—O8	−67.46 (12)	C12—C13—C14—C15	59.55 (13)
N7—C2—C3—C4	175.96 (9)	O19—C14—C15—O20	−64.11 (13)
C1—C2—C3—C4	55.26 (12)	C13—C14—C15—O20	174.33 (10)
O8—C3—C4—O9	−57.58 (13)	O19—C14—C15—C16	63.16 (13)
C2—C3—C4—O9	−177.52 (9)	C13—C14—C15—C16	−58.41 (13)
O8—C3—C4—C5	65.65 (14)	O20—C15—C16—O21	61.04 (13)
C2—C3—C4—C5	−54.29 (13)	C14—C15—C16—O21	−66.54 (13)
O9—C4—C5—O10	55.85 (13)	O20—C15—C16—C17	−178.05 (10)
C3—C4—C5—O10	−64.77 (13)	C14—C15—C16—C17	54.36 (13)
O9—C4—C5—C6	175.88 (9)	O21—C16—C17—N22	−49.61 (13)
C3—C4—C5—C6	55.25 (13)	C15—C16—C17—N22	−173.95 (10)
C2—C1—C6—N11	−178.30 (10)	O21—C16—C17—C12	72.58 (13)
C2—C1—C6—C5	61.17 (13)	C15—C16—C17—C12	−51.77 (13)
O10—C5—C6—N11	−54.54 (13)	N18—C13—C12—C17	179.03 (10)
C4—C5—C6—N11	−177.14 (10)	C14—C13—C12—C17	−58.53 (13)
O10—C5—C6—C1	64.96 (13)	N22—C17—C12—C13	177.03 (10)
C4—C5—C6—C1	−57.64 (13)	C16—C17—C12—C13	54.90 (13)

### Hydrogen-bond geometry (Å, º)

**Table:**

*D*—H···*A*	*D*—H	H···*A*	*D*···*A*	*D*—H···*A*
N18—H18*A*···Cl4	0.89 (1)	2.28 (1)	3.1411 (11)	163 (2)
N18—H18*B*···Cl1^i^	0.90 (1)	2.37 (2)	3.2646 (12)	177 (2)
N18—H18*C*···Cl3^ii^	0.91 (1)	2.26 (1)	3.1634 (11)	175 (2)
N22—H22*C*···Cl4^iii^	0.87 (1)	2.21 (1)	3.0761 (11)	172 (2)
N22—H22*A*···Cl1	0.88 (2)	2.28 (2)	3.1466 (12)	168 (2)
N22—H22*B*···Cl3	0.87 (1)	2.32 (2)	3.1518 (12)	162 (2)
O19—H19···O9^iv^	0.81 (2)	1.91 (2)	2.7168 (13)	172 (2)
O20—H20···Cl2^v^	0.88 (2)	2.48 (2)	3.2220 (10)	143 (2)
O21—H21···Cl2	0.83 (1)	2.39 (2)	3.2096 (10)	174 (2)
N7—H7*C*···O20^vi^	0.89 (1)	2.05 (2)	2.8560 (15)	151 (2)
N7—H7*B*···O19	0.85 (1)	2.11 (2)	2.9404 (14)	164 (2)
N7—H7*A*···Cl3	0.89 (1)	2.70 (2)	3.4038 (11)	138 (1)
O8—H8···Cl2^vi^	0.84 (1)	2.29 (2)	3.1256 (10)	175 (2)
O9—H9···Cl1^iv^	0.80 (2)	2.27 (2)	3.0144 (10)	156 (2)
O10—H10···Cl4^vii^	0.85 (2)	2.14 (2)	2.9889 (10)	177 (2)
N11—H11*C*···Cl2^v^	0.89 (2)	2.37 (2)	3.2132 (12)	159 (2)
N11—H11*B*···Cl1^v^	0.86 (1)	2.51 (2)	3.3007 (11)	154 (2)
N11—H11*A*···Cl2	0.90 (2)	2.61 (2)	3.4618 (13)	158 (2)

Symmetry codes: (i) *x*, *y*+1, *z*; (ii) −*x*+1, *y*+1/2, −*z*; (iii) −*x*+1, *y*−1/2, −*z*; (iv) −*x*+1, *y*+1/2, −*z*+1; (v) −*x*+2, *y*+1/2, −*z*+1; (vi) *x*−1, *y*, *z*; (vii) −*x*+1, *y*−1/2, −*z*+1.
